# Early Screening of Colorectal Precancerous Lesions Based on Combined Measurement of Multiple Serum Tumor Markers Using Artificial Neural Network Analysis

**DOI:** 10.3390/bios13070685

**Published:** 2023-06-27

**Authors:** Xing Ke, Wenxue Liu, Lisong Shen, Yue Zhang, Wei Liu, Chaofu Wang, Xu Wang

**Affiliations:** 1 Department of Pathology, Ruijin Hospital and College of Basic Medical Sciences, Shanghai Jiao Tong University School of Medicine, Shanghai 200025, China; satoshiohno@sjtu.edu.cn (X.K.); qq1727624844@sjtu.edu.cn (W.L.);; 2 Department of Clinical Laboratory, Xinhua Hospital, Shanghai Jiao Tong University School of Medicine, Shanghai 200092, China; lisongshen@hotmail.com; 3 Key Laboratory of Cell Differentiation and Apoptosis of Chinese Ministry of Education, Shanghai Jiao Tong University School of Medicine, Shanghai 200025, China; 4 Institute of Artificial Intelligence Medicine, Shanghai Academy of Experimental Medicine, Shanghai 200092, China; 5 Department of Research Collaboration, R&D Center, Beijing Deepwise & League of PHD Technology Co., Ltd., Beijing 100080, China; 6 Nanning Jiuzhouyuan Biotechnology Co., Ltd., Nanning 530007, China

**Keywords:** colorectal cancer, tumor marker, immunoassay, artificial neural network, early diagnosis

## Abstract

Many patients with colorectal cancer (CRC) are diagnosed in the advanced stage, resulting in delayed treatment and reduced survival time. It is urgent to develop accurate early screening methods for CRC. The purpose of this study is to develop an artificial intelligence (AI)-based artificial neural network (ANN) model using multiple protein tumor markers to assist in the early diagnosis of CRC and precancerous lesions. In this retrospective analysis, 148 cases with CRC and precancerous diseases were included. The concentrations of multiple protein tumor markers (CEA, CA19-9, CA 125, CYFRA 21-1, CA 72-4, CA 242) were measured by electrochemical luminescence immunoassays. By combining these markers with an ANN algorithm, a diagnosis model (CA6) was developed to distinguish between normal healthy and abnormal subjects, with an AUC of 0.97. The prediction score derived from the CA6 model also performed well in assisting in the diagnosis of precancerous lesions and early CRC (with AUCs of 0.97 and 0.93 and cut-off values of 0.39 and 0.34, respectively), which was better than that of individual protein tumor indicators. The CA6 model established by ANN provides a new and effective method for laboratory auxiliary diagnosis, which might be utilized for early colorectal lesion screening by incorporating more tumor markers with larger sample size.

## 1. Introduction

Colorectal cancer (CRC) is one of the leading causes of cancer death in both men and women worldwide [1]. Most CRC patients are diagnosed in the advanced stage because they are usually asymptomatic in the early stage. The five-year survival rate for metastatic CRC is very low, remaining at around 14%, while scientific and clinical advances in early detection and surgery have improved five-year survival rates to 90% and 71% for localized and regionalized CRC, respectively [2]. Given this sobering fact, CRC screening has been recommended in many countries, including China. Therefore, the development of sensitive, efficient, and reliable testing techniques is essential for the early diagnosis of CRC and precancerous lesions, providing more opportunities for effective treatment and intervention.

Currently, widely used clinical methods for CRC screening include endoscopy, stool examination, imaging modalities, and tumor biomarker detection [3]. Colonoscopy, the gold standard for CRC diagnosis, has superior sensitivity for CRC and advanced adenomas (AA), but it is invasive, inconvenient, associated with complications, and more expensive than alternative methods such as the fecal immunochemical test (FIT) and the guaiac fecal occult blood test (gFOBT) [4]. These factors limit the application of endoscopy in early screening for CRC, especially in China, which has an enormous population. In addition, various non-invasive methods have been utilized for early CRC screening, including the FIT, the multi-target fecal DNA (MT-sDNA) test, the plasma DNA methylated *septin* 9 (SEPT9) test, the fecal DNA methylated *syndecan* 2 (SDC2) test, etc., but these methods usually suffer from low sensitivity and specificity for early-stage CRC and precancerous lesions [5,6]. Recently, circulating tumor DNA (ctDNA) analysis using the next generation sequencing (NGS) technique has been utilized for the diagnosis of various cancers, including CRC, and shows good accuracy for late-stage patients [7]. But reliability remains a huge challenge for ctDNA analysis, and the cost of NGS is usually high. Blood biomarker testing as a non-invasive CRC screening strategy has been performed in clinical laboratories for decades and shows great promise in the early diagnosis, prediction, and prognosis of cancer. There have been many studies on the clinical use of protein tumor markers such as serum carcinoembryonic antigen (CEA), carbohydrate antigen 19-9 (CA 19-9), carbohydrate antigen 125 (CA 125), carbohydrate antigen 242 (CA 242), carbohydrate antigen 153 (CA 153), carbohydrate antigen 72-4 (CA 72-4), squamous cell carcinoma (SCC), and cytokeratin-19-fragment (CYFRA 21-1) in the diagnosis of CRC, but the role of these markers in the screening of precancerous lesions and early CRC was rarely investigated [8,9,10,11]. These protein markers are usually measured by an immunological method, in which the target protein is captured by a pair of antibodies in a sandwich format, and the signal is amplified using enzymes or some other labels such as fluorescence or electrochemical substances. Electrochemical luminescence (ECL) immunoassay is one of the most widely used methods for the detection of protein markers in clinics. It has inherent advantages such as good sensitivity and selectivity, simple operation, wide dynamic range, and fast detection speed [12,13]. These tumor marker tests are usually inexpensive and can be performed in most secondary and above medical institutions with reliable results. It is necessary to fully explore the significance of these tumor markers to improve the diagnostic efficiency of precancerous lesions and early CRC so that patients can get early intervention and treatment.

Machine learning (ML) is a subset of artificial intelligence (AI) that has been widely applied for the diagnosis and prognosis assessment of CRC [14,15,16,17,18,19,20]. Previous literature has used different ML models to integrate multiple miRNA hub genes and evaluated their potential to be used as diagnostic panels for CRC [14]. It was also reported that image tiles from patients with different disease outcomes were used to train a total of ten convolutional neural networks specifically for classifying extremely large heterogeneous images [16]. Multilayer perceptron (MLP), also known as artificial neural network (ANN), is a subfield of ML proposed by Rosenblatt in 1957. ANN is the basis of neural networks and support vector machines, focusing on algorithms similar to brain structure and function [21,22]. A model based on ANN is made up of a hierarchy of layers consisting of input, hidden, and output layers. After receiving data, the input layer transmits the data to a hidden layer, which is used for processing the data and subsequently provides results to the output layer. Finally, the output layer shows classification results [23,24]. The reason for choosing an ANN classification algorithm is that it is a robust model that is not complicated. It returns not only the prediction but also the degree of certainty, which is very useful in practical applications.

In this study, we measured the concentration of CRC-related protein markers by ECL immunoassays and established a prediction model using the ANN algorithm. We evaluated the diagnostic performance of this model for precancerous lesions and early CRC. Results indicate that the model not only provides accurate and convenient decision support for the diagnosis of colorectal precancerous lesions and early CRC but also reasonably interprets a large amount of test data, which greatly enhances users’ trust.

## 2. Materials and Methods

### 2.1. Subjects

Prior to starting the study, ethical approval was obtained from the Ethics Committee of Xinhua Hospital, Shanghai Jiao Tong University School of Medicine (Approval No. XHEC-D-2023-093). All cases registered in the Pathology Laboratory of Xinhua Hospital from March 2022 to February 2023 were included in this study with final diagnosis. All cases were divided into two groups: precancerous disease and CRC. Inclusion criteria: (1) precancerous disease: patients with colonoscopy or pathological diagnosis results were included, who were clinically diagnosed with diseases including adenomatous polyp, hyperplastic polyp, and inflammatory polyp. (2) CRC: patients with colorectal adenocarcinoma were enrolled based on clinical and histopathological findings. Exclusion criteria: patients with unclear clinical diagnosis, repeated examination or treatment, or co-existing other malignancies. For patients with precancerous disease or CRC who had been admitted multiple times, only the data for the first diagnosis without treatment (including surgical treatment and drug therapy) was considered to minimize bias.

A total of 148 cases that fulfilled the eligibility criteria were included in this study, including 74 (50%) precancerous lesion cases and 74 (50%) CRC cases. The TNM stage was rescheduled according to the 8th edition *AJCC Cancer Staging Manual* [25]. Clinical guidelines for the diagnosis and treatment of CRC released by the Chinese Society of Clinical Oncology (CSCO) in 2021 defined early-stage CRC as cancer cells confined to the mucous lamina propria or penetrating the musculi of the colorectal mucous membrane to infiltrate into the submucosa but not involving the musculi propria. Of the 74 CRC cases, 18 (24.3%) were in early stage. In order to ensure the accurate comparison of results, 61 apparently healthy people were selected as normal healthy controls during the physical examination.

### 2.2. Measurement of Protein Marker Concentration Using ECL Immunoassay

An amount of 5 mL of venous blood was collected from fasting subjects in the morning and centrifuged at 3000 RPM for 10 min. The serum was separated and stored at −80 °C and thawed immediately prior to testing. The concentrations of serum protein tumor markers (including CEA, CA19-9, CA 125, CYFRA 21-1, CA 72-4, CA 242, CA 153, AFP, and SCC) were measured with an ECL immunoassay analyzer according to Roche’s ECL instruction manual. The total time was 15–25 min. Specifically, the sample was added to a cuvette, and a biotin-labeled capture antibody and a ruthenium complex-labeled detection antibody were subsequently added. The mixture was incubated to form sandwich immune complexes. Then, streptavidin-coated magnetic particles were added to capture the immune complexes via biotin and streptavidin interaction. The reaction mixture was then sucked into the measuring unit, where the particles were magnetically captured to the electrode surface and flushed with tripropylamine and a cleaning solution. Next, the chemiluminescence reaction was performed on the electrode surface, the signal was detected by photomultiplier tubes, and the result was measured by the calibration curves. When the detection result exceeded the detection limit, the high or low value of the detection limit was recorded in the statistics. The indoor quality control, accuracy, precision, and other performance results related to these indicators were acceptable to ensure that the included patients’ data were accurate and reliable.

### 2.3. Development of ANN-Based Prediction Model

All records and observations were reviewed by two board-certified colorectal pathologists. Patients with early-stage CRC accounted for 24.3% (18/74) of all CRC patients. Among the patients with precancerous lesions, adenomatous polyps accounted for 44.6%, hyperplastic polyps accounted for 33.8%, and inflammatory polyps accounted for about 21.6%. Z-score normalization was carried out before modeling, that is, the mean value and standard deviation of the original data were used to normalize the data. The variables that were associated with the patients were used for developing the ANN-based prediction model. In the ANN modeling process, we randomly divided the data into two subsets: 146 patients (nearly 70%) for constructing the model (as the training subset) and the remaining 63 patients (nearly 30%) for testing the model (as the validation subset) (Table 1).

We used the ANN model based on the scikit-learn (sklearn) library for modeling. The architecture of the model includes an input layer, a hidden layer, and an output layer. In the hidden layer, we select 100 neurons and use the rectified linear unit (ReLU) as the activation function. ReLU activation functions perform well in dealing with nonlinear relationships and provide better model representation. We used LBFGS as the solver and set the regularization parameter (alpha) to 0.01 to control the complexity of the model and prevent overfitting. Six markers that could independently distinguish normal healthy and abnormal groups in the receiver operating characteristic (ROC) curve analysis were included in the model (including CEA, CA19-9, CA 125, CYFRA 21-1, CA 72-4, and CA 242). The output of the ANN model was assigned a prediction score between 0 and 1. The higher the output value, the higher the positive risk. Thresholds were then selected from the training set to calculate the specificity and sensitivity of the validation set. We used hyperparameter retrieval to find better parameters of the current model, that is, to find a better feature screening (dimensionality reduction) algorithm and its parameters, so as to obtain better results.

### 2.4. Testing the Performance of ANN-Based Prediction Model

The performance of the AI model based on ANN was evaluated by sensitivity [TP/(TP+FN)], specificity [TN/(TN+FP)], accuracy [TP+TN/(TP+FP+TN+FN)], positive predictive value (PPV) [TP/(TP+FP)], and negative predictive value (NPV) [TN/(TN+FN)]. Matrices of true and predicted conditions are shown in Table S1. In addition, the area under a receiver operation characteristic curve (AUC) was used for comparing the prediction power of the described model. The quantitative index of the prediction ability of the model was directly expressed by the prediction score.

### 2.5. Statistical Analysis

The Deepwise & Beckman Coulter DxAI platform (https://dxonline.deepwise.com/login) (accessed on 26 June 2023) was used to perform the ANN algorithm. This platform was based on scikit-learn 1.2.2 for packaging modeling using the neural network models algorithm. A detailed introduction to the algorithm and the original code can be found on the following website: (https://scikit-learn.org/stable/modules/neural_networks_supervised.html) (accessed on 26 June 2023). Distributed variables were presented as means ± SD, and the significance of differences was determined with Student’s *t*-test or the Wilcoxon rank sum test. The confidence interval (CI) was used to estimate the population parameters of the sample. The chi-square test was analyzed by the SPSS12.0 statistical package. A *p*-value less than 0.05 was considered statistically significant.

## 3. Results

### 3.1. Comparison of Tumor Marker Levels among Different Groups

**Figure 1:**
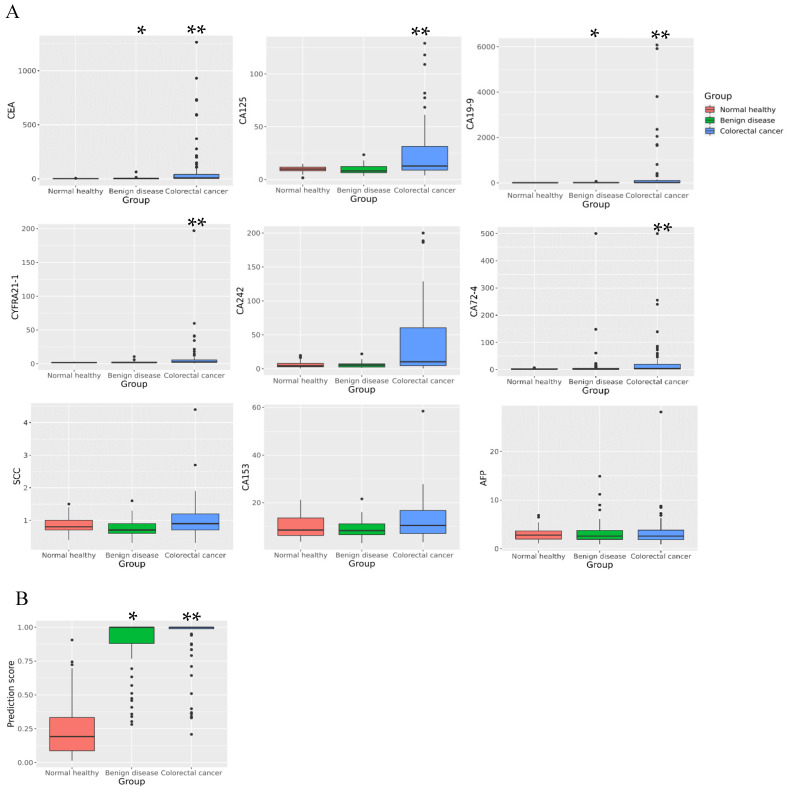
Difference comparison of individual tumor markers and prediction score among the three groups. (**A**) Difference comparison of individual protein tumor markers among the three groups. (**B**) Difference comparison of artificial neural network (ANN)-derived prediction score among the three groups. * *p* < 0.05, the indicators of benign diseases were significantly higher than those of normal healthy subjects. ** *p* < 0.05, the indicators of colorectal cancer (CRC) group were significantly higher than those of normal healthy or benign disease subjects.

Based on the inclusion and exclusion criteria, 209 cases were selected, among which 74 cases were CRC, 74 cases were benign precancerous diseases, and 61 cases were normal healthy controls. The concentrations of protein tumor markers were measured by ECL immunoassays, and the results are shown in Figure 1A. The analytical performance of assays was summarized in Table S2. We compared the levels of nine protein tumor markers among the three groups. The level of CEA [2.7 (95% Cl 1.5–3.7) vs. 1.4 (95% Cl 1.1–2.2)] and CA19-9 [12.3 (95% Cl 7.3–17.4) vs. 7.6 (95% Cl 5.1–10.2)] in benign diseases was significantly higher than that in normal healthy subjects (*p* < 0.001). CEA [7.5 (95% Cl 1.8–40.9) vs. 1.4 (95% Cl 1.1–2.2)], CA19-9 [16.6 (95% Cl 10.3–105.0) vs. 7.6 (95% Cl 5.1–10.2)], CA 72-4 [4.5 (95% Cl 1.6–19.2) vs. 1.5 (95% Cl 1.5–3.1)], CA 242 [10.3 (95% Cl 4.6–60.4) vs. 4.4 (95% Cl 2.9–8.0)], CA 125 [12.8 (95% Cl 8.9–31.4) vs. 9.9 (95% Cl 8.3–11.7)], and CYFRA 21-1 [2.7 (95% Cl 2.0–5.7) vs. 1.7 (95% Cl 1.5–2.2)] in CRC were significantly different from those in normal healthy subjects (*p* < 0.001). When CRC was compared with benign diseases, CEA [7.5 (95% Cl 1.8–40.9) vs. 2.7 (95% Cl 1.5–3.7)], CA19-9 [16.6 (95% Cl 10.3–105.0) vs. 12.3 (95% Cl 7.3–17.4)], CA 125 [12.8 (95% Cl 8.9–31.4) vs. 8.1 (95% Cl 6.5–12.3)], CA 72-4 [4.5 (95% Cl 1.6–19.2) vs. 1.9 (95% Cl 1.5–4.3)], CYFRA 21-1 [2.7 (95% Cl 2.0–5.7) vs. 1.9 (95% Cl 1.2–2.5)], and CA 242 [10.3 (95% Cl 4.6–60.4) vs. 5.4 (95% Cl 2.6–7.3)] showed significant differences (*p* < 0.05).

### 3.2. Evaluation of ANN Model Prediction Efficiency

A total of 209 cases, divided into normal healthy and abnormal groups, were used to train the ANN model based on the parameters mentioned above, including CEA, CA 19-9, CA 125, CA 242, CYFRA 21-1, and CA 72-4. The ANN model, named CA6, finally output the prediction score. The fitting results were satisfactory. The training set had a high accuracy of 94%. The AUC, sensitivity, and specificity of the training set were 0.98, 93%, and 95%, respectively. In the validation set, the prediction results showed that the AUC, accuracy, sensitivity, and specificity were 0.92, 83%, 96%, and 50%, respectively. When the prediction scores of these three groups were compared, the benign disease group was significantly higher than the normal healthy group [1.00 (95% Cl 0.88–1.00) vs. 0.19 (95% Cl 0.09–0.33), *p* < 0.001], and the CRC group was significantly higher than the benign disease group [1.00 (95% Cl 0.99–1.00) vs. 1.00 (95% Cl 0.88–1.00), *p* < 0.05] and the normal healthy group [1.00 (95% Cl 0.99–1.00) vs. 0.19 (95% Cl 0.09–0.33), *p* < 0.001] (Figure 1B).

**Figure 2:**
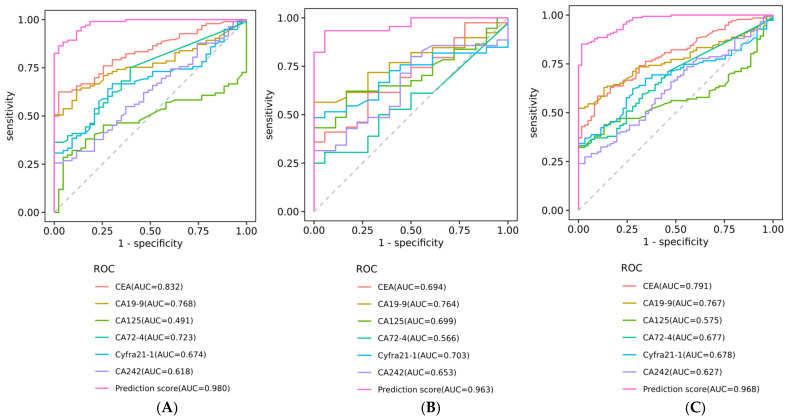
Receiver operating characteristic (ROC) curve of prediction score distinguishing between normal healthy and abnormal group based on six serum tumor markers. (**A**) ROC curve distinguishing between normal healthy and abnormal groups in the training set. (**B**) ROC curve distinguishing between normal healthy and abnormal groups in the validation set. (**C**) ROC curve distinguishing between normal healthy and abnormal groups in the total cases.

When the training set, validation set, and total cases were included as research objects, the ROC curve analysis showed that the CA6 model had a good ability to distinguish between normal healthy and abnormal groups, and the AUC values were 0.98 (95% Cl 0.96–1.00), 0.96 (95% Cl 0.92–1.00) and 0.97 (95% Cl 0.95–0.99), respectively (Table 2, Figure 2A–C).

### 3.3. Diagnostic Efficacy Comparison of ANN Model with Other Markers

By comparing and analyzing the ability to distinguish between the normal healthy group and the abnormal group using the ROC curve, we found that the prediction score was much better than individual tumor markers, and its AUC was 0.97 (95% Cl 0.95–0.99, standard error 0.010, *p* < 0.001). Under the condition of *p* < 0.05, the AUCs of individual tumor markers CEA, CA19-9, CYFRA 21-1, CA 72-4, CA 242, and CA 125 were 0.79 (95% Cl 0.73–0.85), 0.77 (95% Cl 0.70–0.83), 0.68 (95% Cl 0.60–0.76), 0.68 (95% Cl 0.60–0.75), 0.63 (95% Cl 0.54–0.71), and 0.58 (95% Cl 0.49–0.66), respectively. In the training set and validation set, the AUC of prediction score [0.98 (95% Cl 0.96–1.00), standard error 0.008, *p* < 0.001 for training; 0.96 (95% Cl 0.92–1.00), standard error 0.021, *p* < 0.001 for validation] was also significantly higher than that of individual protein tumor markers (Table S3, Figure 2A–C). These results demonstrated that the ANN-derived prediction score was significantly better than individual protein tumor indicators in distinguishing between normal healthy and abnormal subjects.

### 3.4. Consistency between ANN Model and Clinical Diagnosis

**Figure 3:**
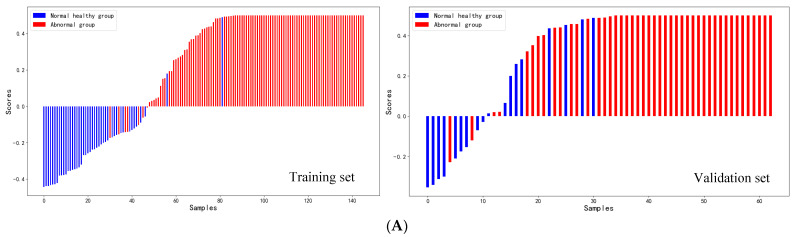

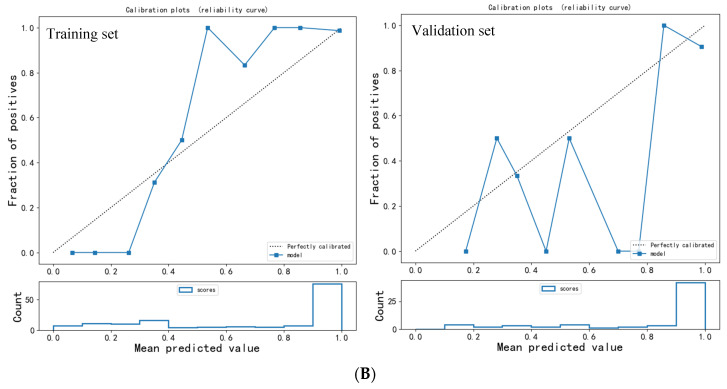
Consistency analysis between the model prediction score and clinical diagnosis based on the CA6 model. (**A**) Model scoring graph of CA6 model in training set and validation set. (**B**) Calibration curve of CA6 model in training set and validation set.

The agreement between the prediction behavior of CA6 model and the actual diagnosis was further analyzed. In both the training and validation sets, patients’ risk scores were consistent with clinical diagnosis results (*p* < 0.05) (Figure 3A). The histogram in the upper part of Figure 3A was marked with different colors to indicate that some subjects predicted by the model to be abnormal were actually normal in pathological diagnosis (false positive: 2/98 in training and 9/52 in validation). In contrast, the bottom histogram shows that some subjects with abnormal pathological diagnosis were predicted to be normal by the model (false negative: 7/48 in training and 2/11 in validation). Results demonstrate that the false positive rate and false negative rate of the CA6 model were lower than those of conventional tumor markers. As can be seen from the calibration curve (Figure 3B), the predicted value of the model was close to the actual diagnosis probability. The calibration curve is an evaluation index suitable for probabilistic models such as ANN. It isa curve with the predicted value as the abscissa and the real value as the ordinate. The closer the calibration curve was to the diagonal, the better the performance of the model. The consistency of the total number of normal healthy group and abnormal group patients predicted by the training set and validation set was compared with pathological diagnosis results, and the chi-square test showed a good consistency (χ^2^ = 107.794, *p* < 0.001 and χ^2^ = 18.515, *p* < 0.001) (Table 3). These results showed that the prediction results of the CA6 model are in good agreement with the actual diagnosis.

### 3.5. Evaluation of ANN Model Prediction Efficiency in Early Colorectal Diseases

**Figure 4:**
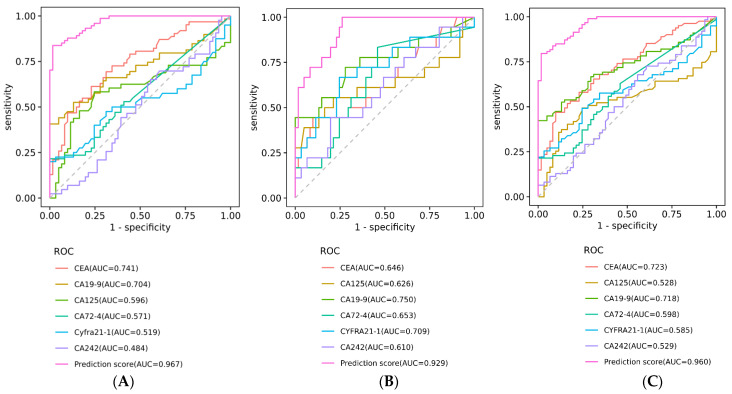
Receiver operating characteristic (ROC) curve of CA6 mode to distinguish benign disease and early-stage colorectal cancer (CRC) from normal healthy people. (**A**) ROC curve distinguishing between benign disease and normal healthy people. (**B**) ROC curve distinguishing between early-stage CRC and normal healthy people. (**C**) ROC curve distinguishing benign disease and early-stage CRC from normal healthy people.

In order to evaluate the diagnostic efficiency of the CA6 model for benign diseases and early-stage CRC, 74 cases of benign diseases and 18 cases of early-stage CRC were analyzed. The CA6 model had high diagnostic efficiency in distinguishing between normal healthy subjects and patients with benign disease or early-stage CRC. Forthe benign disease, it had an AUC of 0.97 (95% Cl 0.94–0.99). When the cut-off value was set to 0.39, the specificity was 80%, and the sensitivity was 94% (Table 4 and Table S4). The diagnosis efficiency of early-stage CRC was also good, with an AUC of 0.93 (95% Cl 0.87–0.97), a cut-off value of 0.34, a specificity of 75%, and a sensitivity of 94%. The AUC of the prediction score of benign disease and early CRC was significantly higher than that of individual protein tumor indicators (Figure 4 and Table S4). When the cases of benign disease and early CRC were pooled together, the overall prediction score was also significantly higher than that of individual protein tumor indicators; it had an AUC of 0.96 (95% Cl 0.94–0.99), a cut-off value of 0.30, a specificity of 72%, and a sensitivity of 97%. The number of patients with normal and early colorectal disease identified by the AUC curve was compared with the actual diagnosis, and the chi-square test showed good agreement (χ^2^ = 89.172, *p* < 0.001) (Table 5). These results illustrated that the CA6 model established by the ANN algorithm had a great potential diagnostic efficacy not only for total abnormal subjects but also for benign disease and early-stage CRC, which is of great significance for improving the clinical recognition of early colorectal diseases.

## 4. Discussion

Many CRC patients have no symptoms or signs in the early stage and thus cannot be diagnosed and treated in time. Delayed diagnosis and treatment significantly reduce patient survival time. CRC is mainly diagnosed by endoscopy and pathological biopsy combined with clinical symptoms [26]. Although these methods are effective, they are invasive, which might cause harm to patients and are thus not suitable for dynamic monitoring of disease. Non-invasive methods, such as fecal hemoglobin, which are used for large-scale screening, have been shown to reduce CRC-related mortality [27,28]. These methods have advantages with respect to cost, safety, and convenience [29]. However, they usually suffer from low sensitivity and specificity and might cause false positive results if the subjects are not compliant with screening recommendations. In addition, fecal or plasma DNA methylation tests, NGS tests of ctDNA, etc. all have their own shortcomings, such as low specificity and high cost. Current diagnostic methods for the diagnosis of early colorectal lesions, especially precancerous lesions, are not satisfactory. Therefore, it is important to develop safe, reliable, specific, effective methods for the accurate diagnosis of early colorectal lesions. Previous studies have demonstrated that analysis of multiple tumor markers combined with ML technology provides a promising platform for cancer diagnosis [30]. Therefore, we measured blood concentrations of multiple protein markers that are relevant to the diagnosis, disease surveillance, or prognosis of gastrointestinal tumors. The ANN algorithm was used to establish a model to distinguish between healthy and unhealthy subjects and to verify the efficiency of the model in diagnosing early colorectal diseases.

ECL immunoassay has been widely used for the detection of various clinical protein markers due to its high automaticity, easy operation process, accurate results, and good traceability. Those markers detected by the ECL method have been well used in the diagnosis, disease monitoring, and prognosis of various cancers. Serum tumor markers are used for the diagnosis of CRC, but single markers usually show insufficient sensitivity and specificity. In this study, we tested nine protein markers, among which CEA and CA19-9 were recommended in the Chinese expert consensus on experimental diagnostic technology for screening of early CRC and precancerous lesions [31]. CA125 was a cell surface glycoprotein that is abnormally expressed in most gastrointestinal adenocarcinomas and associated with the diagnosis and prognosis of CRC. CA 72-4 and CA 242 were often recommended as complementary indicators for screening and monitoring the therapeutic efficacy of gastrointestinal tumors [32]. In recent studies, CYFRA 21-1 was integrated with some other markers for CRC identification, although it was generally considered as a marker for non-small cell lung cancer [33]. Therefore, we focused on the role of these tumor markers in the early diagnosis of colorectal lesions.

Combining multiple serum tumor markers increases diagnostic sensitivity but decreases specificity, and vice versa. The development of AI and computer technology improves the potential of multiple serum tumor markers in screening for certain diseases [34]. In this study, a popular data mining algorithm, ANN, was used. ANN has the advantage of automatically detecting and modeling complex nonlinear relationships between the input layer and the output layer of the network and contains all possible interactions among input variables. The layers used in the ANN algorithm are composed of interconnected neurons. ANN analysis as a statistical modeling tool that has demonstrated the ability to assimilate information from multiple sources and detect subtle and complex patterns [35]. Recently, a large number of studies have discussed the application of ANN in cancer diagnosis and treatment guidance. Matsuda et al. used ANN to evaluate the endoscopic response of esophageal cancer patients receiving neoadjuvant chemotherapy [36]. Fan et al. developed a non-invasive and low-cost artificial neural network model integrating CA125, AFP, and CA242 tests, which was a valuable tool to assist in the diagnosis of gastric cancer [22]. Liu et al. demonstrated that their risk score model was robust and reliable for evaluating the prognosis with novel diagnostic and treatment targets in CRC [37]. Abdul Rahman et al. trained ANN models with large heterogeneous datasets and provided a solid foundation for building effective clinical decision support systems assisting healthcare providers in dietary-related, non-invasive screening in CRC [38].

Although there are many factors in CRC diagnosis, and the relationship between them is complex, ANN can learn fuzzy evaluation that cannot be described by mathematical methods and deal with some complex, uncertain, and nonlinear problems by imitating human intelligent behavior, which has good fault tolerance and fast parallel processing ability. Based on the ANN algorithm, multiple serum tumor markers were combined for modeling. Our analysis showed that the ANN model can improve the diagnostic efficiency of colorectal diseases, including benign lesions and CRC. In the ROC curve analysis of both the training set and the validation set, the prediction score output by ANN was proven to be superior to individual markers such as CEA and CA19-9. When the levels of the ANN-derived prediction score among these three groups were compared, the benign disease group was significantly higher than the normal healthy group, while the CRC group was significantly higher than the benign disease and normal healthy groups. These results indicate that the prediction score was also an important indicator for these three groups.

Importantly, the prediction score significantly improved the AUC, sensitivity, specificity, and accuracy of the diagnosis potential of benign precancerous lesions or early CRC, i.e., ANN can be used to distinguish benign lesions or early CRC from healthy people. Comparative studies of ROC curves support the conclusion that the ANN model using multiple markers improves sensitivity and has higher diagnostic accuracy without sacrificing specificity. ANN can be used to improve the accuracy of combined diagnosis of multiple serum tumor markers. Prediction score combined with six serum tumor markers can distinguish not only benign diseases but also early CRC from normal controls. This strong evidence proves that ANN model is a promising tool to assist in the diagnosis and screening of early CRC. Based on the characteristics of good accuracy and low cost of the ANN model, it is expected to be used as an intelligent tool to screen the high-risk for CRC population for primary prevention. Large prospective cohort studies can further determine whether individuals identified as part of the high-risk group by the ANN model will be diagnosed as CRC in subsequent years. In addition, new serum markers need to be included to develop practical and reliable ANN models to assess the risk of CRC.

## 5. Conclusions

We measured CRC-related protein tumor markers by ECL immunoassays and constructed model CA6 for early diagnosis of colorectal lesions using the ANN algorithm. This model integrated six protein tumor marker variables. The diagnostic efficiency of this model was satisfactory, and it could significantly improve the ability to distinguish early-stage CRC and precancerous lesions from normal healthy people. Results demonstrate that a diagnosis model integrating multiple tumor marker data is very useful in enhancing laboratory auxiliary diagnosis and prediction. Further studies with more tumor markers and a larger population are required to make the model more accurate and reliable.

## Figures and Tables

**Table 1:** Clinical information of subjects.

Group	Feature	Training Set	Validation Set	Total
Colorectal cancer	n	51	23	74
	Age (years), mean ± SD	65 ± 11	65 ± 11	65 ± 11
	Gender			
	Male	27	12	39
	Female	24	11	35
	Clinical stage			
	Early stage	12	6	18
	Advanced stage	39	17	56
Benign disease	n	52	22	74
	Age (years), mean ± SD	62 ± 10	63 ± 10	62 ± 10
	Gender			
	Male	28	13	41
	Female	24	9	33
	Pathological classification			
	Adenomatous polyp	22	11	33
	Hyperplastic polyp	18	7	25
	Inflammatory polyp	12	4	16
Normal healthy control	n	42	19	61
	Age (years), mean ± SD	58 ± 12	59 ± 12	58 ± 12
	Gender			
	Male	21	9	30
	Female	21	10	31
Total	\	94	41	135

**Table 2:** Diagnostic efficiency of prediction score distinguishing between normal healthy and abnormal groups.

Set	Index	AUC	Standard Error	95% CI	*p*
Training set	Prediction score	0.98	0.008	0.96–1.00	0.000 **
Validation set	Prediction score	0.96	0.021	0.92–1.00	0.000 **
Total cases	Prediction score	0.97	0.010	0.95–0.99	0.000 **

** p < 0.001, the difference was statistically significant. AUC: area under the receiver operating characteristic curve; CI: confidence interval.

**Table 3:** Chi-square test of ANN model.

Set	Label Class	Normal Healthy	Abnormal Group	χ^2^	*p*
Training	Normal healthy group	41	2	107.794	0.000 **
	Abnormal group	7	96		
Validation	Normal healthy group	9	9	18.515	0.000 **
	Abnormal group	2	43		

** *p* < 0.001, the difference was statistically significant.

**Table 4:** Diagnostic efficiency of ANN and serum tumor markers to distinguish benign diseases and early-stage colorectal cancer (CRC) from normal healthy subjects.

Group	Index	AUC	Standard Error	95% CI	*p*	Cut-Off	Sensitivity	Specificity	PPV	NPV
Benign disease	Prediction score	0.97	0.012	0.94–0.99	0.000 **	0.39	94%	80%	83%	93%
Early-stage CRC	Prediction score	0.93	0.029	0.87–0.99	0.000 **	0.34	0.94%	75%	79%	93%
Benign disease and early-stage CRC	Prediction score	0.96	0.013	0.94–0.99	0.000 **	0.30	0.97%	72%	78%	96%

** *p* < 0.001, the difference was statistically significant. AUC: area under the receiver operating characteristic curve; CI: confidence interval; PPV: positive predictive value; NPV: negative predictive value.

**Table 5:** Chi-square test of CA6 model for prediction of early colorectal diseases.

Label Class	Normal Healthy	Benign Disease & Early CRC	χ^2^	*p*
Normal healthy	44	17	89.172	0.000 **
Benign disease & Early CRC	1	91		

** *p* < 0.001, the difference was statistically significant. CRC: colorectal cancer.

## Data Availability

The data presented in this study are available on request.

## Supplementary Materials

The following supporting information can be downloaded at: https://www.mdpi.com/article/10.3390/bios13070685/s1, Table S1. Matrices of true and predicted conditions. Table S2. Electrochemical luminescence performance of nine tumor markers. Table S3. Diagnostic efficiency of univariate marker to distinguish between normal healthy and abnormal groups. Table S4. Diagnostic efficiency of univariate marker to distinguish benign diseases and early-stage colorectal cancer (CRC) from normal healthy subjects.

## References

[1] Chuang J.P., Tsai H.L., Chen P.J., Chang T.K., Su W.C., Yeh Y.S., Huang C.W., Wang J.Y. (2022). Comprehensive Review of Biomarkers for the Treatment of Locally Advanced Colon Cancer. Cells.

[2] Lichtenstern C.R., Ngu R.K., Shalapour S., Karin M. (2020). Immunotherapy, Inflammation and Colorectal Cancer. Cells.

[3] Zhang X., Tan X., Wang P., Qin J. (2023). Application of Polypyrrole-Based Electrochemical Biosensor for the Early Diagnosis of Colorectal Cancer. Nanomaterials.

[4] Ribe S.G., Botteri E., Løberg M., Randel K.R., Kalager M., Nilsen J.A., Gulichsen E.H., Holme Ø. (2023). Impact of time between faecal immunochemical tests in colorectal cancer screening on screening results: A natural experiment. Int. J. Cancer.

[5] Alustiza M., Ripoll L., Canals A., Murcia O., Martínez-Roca A., García-Heredia A., Giner-Calabuig M., Jover R., Vidal L. (2023). A novel non-invasive colorectal cancer diagnostic method: Volatile organic compounds as biomarkers. Clin. Chim. Acta.

[6] Yuan R.Q., Zhao H., Wang Y., Song K., Yang J., He W., Miao D.Z., Wang Q., Jia Y.H. (2022). SEPTIN9-SDC2-VIM methylation signature as a biomarker for the early diagnosis of colorectal cancer. Am. J. Cancer Res..

[7] Qi C., Zhou T., Bai Y., Chen H., Yuan J., Zhao F., Liu C., Ma M., Bei T., Chen S. (2023). China Special Issue on Gastrointestinal Tumor-*NTRK* Fusion in a Large Real-World Population and Clinical Utility of Circulating Tumor DNA Genotyping to Guide TRK Inhibitor treatment. Int. J. Cancer.

[8] Cao H., Zhu L., Li L., Wang W., Niu X. (2023). Serum CA724 Has No Diagnostic Value for Gastrointestinal Tumors. Clin. Exp. Med..

[9] Paku M., Uemura M., Kitakaze M., Miyoshi N., Takahashi H., Mizushima T., Doki Y., Eguchi H. (2023). Clinical Significance of Preoperative and Postoperative Serum CEA and Carbohydrate Antigen 19-9 Levels in Patients Undergoing Curative Resection of Locally Recurrent Rectal Cancer. Dis. Colon Rectum.

[10] Luo H., Shen K., Li B., Li R., Wang Z., Xie Z. (2020). Clinical significance and diagnostic value of serum NSE, CEA, CA19-9, CA125 and CA242 levels in colorectal cancer. Oncol. Lett..

[11] Yajima S., Suzuki T., Oshima Y., Shiratori F., Funahashi K., Kawai S., Nanki T., Muraoka S., Urita Y., Saida Y. (2021). New Assay System Elecsys Anti-p53 to Detect Serum Anti-p53 Antibodies in Esophageal Cancer Patients and Colorectal Cancer Patients: Multi-institutional Study. Ann. Surg. Oncol..

[12] Zanut A., Fiorani A., Canola S., Saito T., Ziebart N., Rapino S., Rebeccani S., Barbon A., Irie T., Josel H.P. (2020). Insights into the mechanism of coreactant electrochemiluminescence facilitating enhanced bioanalytical performance. Nat. Commun..

[13] Lippi G., Salvagno G.L., Pegoraro M., Militello V., Caloi C., Peretti A., De Nitto S., Bovo C., Lo Cascio G. (2020). Preliminary evaluation of Roche Cobas Elecsys Anti-SARS-CoV-2 chemiluminescence immunoassay. Clin. Chem. Lab. Med..

[14] Koppad S., Basava A., Nash K., Gkoutos G.V., Acharjee A. (2022). Machine Learning Based Identification of Colon Cancer Candidate Diagnostics Genes. Biology.

[15] Du G., Ren C., Wang J., Ma J. (2022). The Clinical Value of Blood miR-654-5p, miR-126, miR-10b, and miR-144 in the Diagnosis of Colorectal Cancer. Comput. Math. Methods Med..

[16] Skrede O.J., De Raedt S., Kleppe A., Hveem T.S., Liestøl K., Maddison J., Askautrud H.A., Pradhan M., Nesheim J.A., Albregtsen F. (2020). Deep learning for prediction of colorectal cancer outcome: A discovery and validation study. Lancet.

[17] Kavitha M.S., Gangadaran P., Jackson A., Venmathi Maran B.A., Kurita T., Ahn B.C. (2022). Deep Neural Network Models for Colon Cancer Screening. Cancers.

[18] Niehues J.M., Quirke P., West N.P., Grabsch H.I., van Treeck M., Schirris Y., Veldhuizen G.P., Hutchins G.G.A., Richman S.D., Foersch S. (2023). Generalizable biomarker prediction from cancer pathology slides with self-supervised deep learning: A retrospective multi-centric study. Cell Rep. Med..

[19] Chan H.C., Chattopadhyay A., Chuang E.Y., Lu T.P. (2021). Development of a Gene-Based Prediction Model for Recurrence of Colorectal Cancer Using an Ensemble Learning Algorithm. Front. Oncol..

[20] Wang L. (2022). Predicting Colorectal Cancer Using Residual Deep Learning with Nursing Care. Contrast Media Mol. Imaging.

[21] Stulp F., Sigaud O. (2015). Many regression algorithms, one unified model: A review. Neural Netw..

[22] Fan Z., Guo Y., Gu X., Huang R., Miao W. (2022). Development and validation of an artificial neural network model for non-invasive gastric cancer screening and diagnosis. Sci. Rep..

[23] Nasser M., Yusof U.K. (2023). Deep Learning Based Methods for Breast Cancer Diagnosis: A Systematic Review and Future Direction. Diagnostics.

[24] Wang Y., Qian H., Shao X., Zhang H., Liu S., Pan J., Xue W. (2023). Multimodal convolutional neural networks based on the Raman spectra of serum and clinical features for the early diagnosis of prostate cancer. Spectrochim. Acta A Mol. Biomol. Spectrosc..

[25] Amin M.B., Greene F.L., Edge S.B., Compton C.C., Gershenwald J.E., Brookland R.K., Meyer L., Gress D.M., Byrd D.R., Winchester D.P. (2017). The Eighth Edition AJCC Cancer Staging Manual: Continuing to build a bridge from a population-based to a more “personalized” approach to cancer staging. CA Cancer J. Clin..

[26] Maes-Carballo M., García-García M., Martín-Díaz M., Estrada-López C.R., Iglesias-Álvarez A., Filigrana-Valle C.M., Khan K.S., Bueno-Cavanillas A. (2023). A comprehensive systematic review of colorectal cancer screening clinical practices guidelines and consensus statements. Br. J. Cancer.

[27] Hanna M., Dey N., Grady W.M. (2023). Emerging Tests for Noninvasive Colorectal Cancer Screening. Clin. Gastroenterol. Hepatol..

[28] Ding Q., Kong X., Zhong W., Liu W. (2022). Fecal biomarkers: Non-invasive diagnosis of colorectal cancer. Front. Oncol..

[29] Shaukat A., Levin T.R. (2022). Current and future colorectal cancer screening strategies. Nat. Rev. Gastroenterol. Hepatol..

[30] Swanson K., Wu E., Zhang A., Alizadeh A.A., Zou J. (2023). From patterns to patients: Advances in clinical machine learning for cancer diagnosis, prognosis, and treatment. Cell.

[31] Gao Y., Wang J., Zhou Y., Sheng S., Qian S.Y., Huo X. (2018). Evaluation of Serum CEA, CA19-9, CA72-4, CA125 and Ferritin as Diagnostic Markers and Factors of Clinical Parameters for Colorectal Cancer. Sci. Rep..

[32] Liu S., Xu M., Qiao X., Ji C., Li L., Zhou Z. (2021). Prediction of serosal invasion in gastric cancer: Development and validation of multivariate models integrating preoperative clinicopathological features and radiographic findings based on late arterial phase CT images. BMC Cancer.

[33] Gawel S.H., Lucht M., Gomer H., Treado P., Christensen I.J., Nielsen H.J., Davis G.J. (2019). Danish Research Group on Early Detection of Colorectal Cancer.Evaluation of algorithm development approaches: Development of biomarker panels for early detection of colorectal lesions. Clin. Chim. Acta.

[34] Wang L., Zhang M., Pan X., Zhao M., Huang L., Hu X., Wang X., Qiao L., Guo Q., Xu W. (2022). Integrative Serum Metabolic Fingerprints Based Multi-Modal Platforms for Lung Adenocarcinoma Early Detection and Pulmonary Nodule Classification. Adv. Sci..

[35] Dihge L., Bendahl P.O., Skarping I., Hjärtström M., Ohlsson M., Rydén L. (2023). The implementation of NILS: A web-based artificial neural network decision support tool for noninvasive lymph node staging in breast cancer. Front. Oncol..

[36] Matsuda S., Irino T., Kawakubo H., Takeuchi M., Nishimura E., Hisaoka K., Sano J., Kobayashi R., Fukuda K., Nakamura R. (2023). Evaluation of Endoscopic Response Using Deep Neural Network in Esophageal Cancer Patients Who Received Neoadjuvant Chemotherapy. Ann. Surg. Oncol..

[37] Liu Z., Georgakopoulos-Soares I., Ahituv N., Wong K.C. (2023). Risk scoring based on DNA methylation-driven related DEGs for colorectal cancer prognosis with systematic insights. Life Sci..

[38] Abdul Rahman H., Ottom M.A., Dinov I.D. (2023). Machine learning-based colorectal cancer prediction using global dietary data. BMC Cancer.

