# Supplementary material for: Early Screening of Colorectal Precancerous Lesions Based on Combined Measurement of Multiple Serum Tumor Markers Using Artificial Neural Network Analysis
Source: Biosensors (Basel). 2023 Jun 27;13(7):685. doi: 10.3390/bios13070685 (PMC10377288; doi:10.3390/bios13070685)
Supplement: Supplementary file 1 [file biosensors-13-00685-s001.zip › biosensors-2398160-supplementary.pdf]

Supplementary Material

Early Screening of Colorectal Precancerous Lesions Based on Combined Measurement of Multiple Serum Tumor Markers Using Artificial Neural Network Analysis

Xing Ke, Wenxue Liu, Lisong Shen, Yue Zhang, Wei Liu, Chaofu Wang and Xu Wang

Table S1. Matrices of true and predicted condition.

True condition	Predicted condition	
	Predicted positive	Predicted negative
Condition positive	True positive (TP)	False negative (FN)
Condition negative	False positive (FP)	True negative (TN)

Table S2. Electrochemical luminescence performance of nine tumor markers.

Index	Diagnostic threshold	Measuring range	LOB	LOD	LOQ	Unit
CEA	5.2	0.3-1000	0.3	0.6	1.8	ng/ml
CA19-9	39	2-1000	1.5	2	9	U/ml
CA72-4	6.9	1.5-250	0.75	1.5	2.5	U/ml
Cyfra21-1	3.3	0.1-500	0.1	0.3	0.5	ng/ml
CA242	20	0.5 - 200	0.5	1.5	2	U/ml
CA125	35	0.6-5000	0.6	1.2	2.0	U/ml
AFP	5.8	0.75-1000	0.75	1.5	2.25	IU/ml
CA153	25	1.5-300	1	1.5	3	U/ml
SCC	1.5	0.1-70	0.1	0.2	0.6	ng/ml

LOB: Limit of blank; LOD: Limit of detection; LOQ: Limit of quantitation.

Table S3. Diagnostic efficiency of univariate marker to distinguish between normal healthy and abnormal groups.

**Set**	**Index**	**AUC**	**Standard error**	**95% CI**	***P***
Training set	CEA	0.83	0.033	0.77-0.90	0.000 **
	CA19-9	0.77	0.039	0.69-0.85	0.000 **
	CA72-4	0.72	0.043	0.64-0.81	0.000 **
	Cyfra21-1	0.67	0.048	0.58-0.77	0.001 **
	CA242	0.62	0.051	0.52-0.72	0.015 *
	CA153	0.57	0.056	0.46-0.68	0.098
	AFP	0.49	0.052	0.39-0.60	0.458
	CA125	0.49	0.051	0.39-0.59	0.438
	SCC	0.44	0.058	0.33-0.56	0.839
Validation set	CA19-9	0.76	0.062	0.64-0.89	0.001 **
	Cyfra21-1	0.70	0.073	0.56-0.85	0.009 **
	CA125	0.70	0.070	0.56-0.84	0.009 **
	CEA	0.69	0.071	0.56-0.83	0.010 **
	CA242	0.65	0.077	0.50-0.80	0.036 *
	CA72-4	0.57	0.077	0.42-0.72	0.214
	SCC	0.55	0.097	0.36-0.74	0.298
	AFP	0.51	0.080	0.36-0.67	0.565
	CA153	0.48	0.085	0.36-0.65	0.419
Total cases	CEA	0.79	0.032	0.73-0.85	0.000 **
	CA19-9	0.77	0.033	0.70-0.83	0.000 **
	CYFRA21-1	0.68	0.040	0.60-0.76	0.000 **
	CA72-4	0.68	0.038	0.60-0.75	0.000 **
	CA242	0.63	0.042	0.54-0.71	0.003 **
	CA125	0.58	0.042	0.49-0.66	0.049 *
	CA153	0.56	0.046	0.47-0.65	0.112
	AFP	0.50	0.043	0.42-0.59	0.503
	SCC	0.47	0.049	0.38-0.57	0.704

*P<0.05, **P<0.001, the difference was statistically significant. AUC: area under the receiver operating characteristic curve; CI: confidence interval.

Table S4. Diagnostic efficiency of univariate marker to distinguish benign diseases and early-stage colorectal cancer (CRC) from normal healthy subjects.

Group	Index	AUC	Standard error	95% CI	*P*	Cut-off	Sensitivity	Specificity	PPV	NPV
Benign disease	CEA	0.74	0.045	0.65-0.83	0.000 **	1.66	71%	66%	67%	70%
	CA19-9	0.70	0.049	0.61-0.80	0.000 **	12.15	53%	87%	80%	65%
	SCC	0.62	0.064	0.49-0.74	0.969					
	CA125	0.60	0.060	0.48-0.71	0.957					
	CA72-4	0.57	0.052	0.47-0.67	0.084					
	Cyfra21-1	0.52	0.065	0.39-0.65	0.377					
	CA153	0.51	0.059	0.40-0.63	0.581					
	AFP	0.50	0.054	0.39-0.60	0.465					
	CA242	0.48	0.058	0.37-0.60	0.609					
Early-stage CRC	CA19-9	0.75	0.078	0.60-0.90	0.001 **	9.86	72%	72%	72%	72%
	CYFRA21-1	0.71	0.079	0.55-0.86	0.004 **	2.19	67%	75%	73%	69%
	CA72-4	0.65	0.073	0.51-0.80	0.020 *	1.52	83%	54%	65%	76%
	CEA	0.65	0.082	0.49-0.81	0.031 *	2.83	44%	87%	77%	61%
	SCC	0.75	0.103	0.54-0.95	0.988					
	CA125	0.63	0.095	0.44-0.81	0.054					
	CA242	0.61	0.078	0.46-0.76	0.079					
	AFP	0.57	0.077	0.42-0.72	0.817					
	CA153	0.53	0.083	0.37-0.69	0.346					
Benign disease and early-stage CRC	CEA	0.72	0.042	0.64-0.81	0.000 **	0.26	62%	77%	73%	67%
	CA19-9	0.72	0.043	0.63-0.80	0.000 **	9.64	73%	71%	71%	72%
	SCC	0.65	0.057	0.54-0.76	0.995	0.75	43%	31%	39%	35%
	CA72-4	0.60	0.047	0.51-0.69	0.022 *	1.73	54%	59%	57%	56%
	CYFRA21-1	0.59	0.054	0.48-0.69	0.055					
	CA242	0.53	0.053	0.43-0.63	0.29					
	CA125	0.53	0.054	0.42-0.63	0.706					
	AFP	0.52	0.050	0.42-0.61	0.617					
	CA153	0.50	0.053	0.39-0.60	0.494					

*P<0.05, **P<0.001, the difference was statistically significant. AUC: area under the receiver operating characteristic curve; CI: confidence interval; PPV: positive predictive value; NPV: negative predictive value.
